# The Co-Occurrence of 22q11.2 Deletion Syndrome and Epithelial Basement Membrane Dystrophy: A Case Report and Review of the Literature

**DOI:** 10.3390/life14081006

**Published:** 2024-08-13

**Authors:** Marta Armentano, Ludovico Alisi, Francesca Giovannetti, Valeria Iannucci, Luca Lucchino, Alice Bruscolini, Alessandro Lambiase

**Affiliations:** Department of Sense Organs, Sapienza University of Rome, 00185 Rome, Italy; marta.armentano@uniroma1.it (M.A.); ludovico.alisi@uniroma1.it (L.A.); francesca.giovannetti@uniroma1.it (F.G.); valeria.iannucci@uniroma1.it (V.I.); luca.lucchino@uniroma1.it (L.L.); alessandro.lambiase@uniroma1.it (A.L.)

**Keywords:** confocal microscopy, DiGeorge syndrome, 22q11.2 deletion syndrome, map-dot-fingerprint dystrophy, ocular rare diseases

## Abstract

Background: 22q11.2 deletion syndrome (22q11.2DS) is a genetic disorder caused by the deletion of the q11.2 band of chromosome 22. It may affect various systems, including the cardiovascular, immunological, gastrointestinal, endocrine, and neurocognitive systems. Additionally, several ocular manifestations have been described. Results: We report a case of a 34-year-old female diagnosed with 22q11.2DS who presented with visual discomfort and foreign body sensation in both eyes. She had no history of recurrent ocular pain. A comprehensive ophthalmological examination was performed, including anterior segment optical coherence tomography and in vivo confocal microscopy. Overall, the exams revealed bilateral corneal map-like lines, dots, and fingerprint patterns, consistent with a diagnosis of epithelial basement membrane dystrophy (EBMD). In addition to presenting with this novel corneal manifestation for 22q11.2 DS, we review the ocular clinical features of 22q11.2DS in the context of our case. Conclusions: The EBMD may represent a new corneal manifestation associated with 22q11.2 syndrome, although the link between these conditions is unknown. Further research is warranted to investigate potentially shared genetic or molecular pathways to the understanding of the phenotypic variety observed among this rare syndrome.

## 1. Introduction

22q11.2 deletion syndrome (22q11.2DS) is the most common microdeletion human disorder, caused by meiotic chromosome rearrangements. This condition affects approximately 1 in 4000 live births, regardless of sex and ethnic background [1,2]. Although 22q11.2DS is an autosomal dominant condition, more than 90% of patients have unaffected parents, resulting from sporadic microdeletions in the q11.2 region of chromosome 22 [1,3]. It is well known that the disease is caused by microdeletions ranging from 0.7 to 3 million base pairs in size, most of the time included between the low copy number repeats (LCRs) A-D. Within this region, over 90 genes are found, with T-box 1 (TBX1) being the most studied [4]. TBX1 is involved in a myriad of developmental pathways, is expressed in all three germ layers, and is responsible for the communications between the three germ layers and the neural crest [5,6]. Many of the physical abnormalities commonly seen in individuals with 22q11.2DS result from issues in the development and functioning of structures originating from the pharyngeal arch system. This includes craniofacial features, the thymus, parathyroid glands, aortic arch, and cardiac outflow tract. These structures are formed from a combination of cells from the three primary germ layers of the embryo (endoderm, mesoderm, and ectoderm) as well as neural crest cells originating from the closing neural tube [4]. Currently, despite the relatively common occurrence of ocular manifestations in 22q11.2DS, there is a significant lack of information about the genetic determinants of such alterations [7].

Clinically, it manifests with extremely varied symptoms, grouped into different phenotypes defined as DiGeorge syndrome, velocardiofacial (Shprintzen) syndrome, and conotruncal anomaly face (Takao) syndrome [8,9]. These disorders present a variable expressivity and heterogeneous presentation. The most common manifestations are congenital cardiovascular disease (74%), predominantly conotruncal heart malformations; facial dysmorphia and palatal dysfunction (69%); immune deficiency, especially thymic hypoplasia (77%); and endocrinopathies (50%), including thyroid dysfunction, hypoparathyroidism with resultant hypocalcemia, and growth hormone deficiency [2,10,11]. Moreover, affected individuals typically exhibit neurodevelopmental and behavioral disorders such as cognitive delay, learning difficulties (70–90%), attention-deficit hyperactivity disorder (ADHD), and schizophrenia [12,13,14,15]. The diagnostic suspicion is based on clinical evaluation. In particular, the finding of specific cardiac anomalies, such as Fallot tetralogy; truncus arteriosus; ventricular abnormalities; right aortic arch or interrupted aortic arch, detected by echographic examinations in association with facial; palatal; and vertebral bone dysmorphisms, is very suggestive. The definitive diagnosis is established by genomic analysis to confirm the chromosomal deletion performing fluorescence in situ hybridization (FISH) or Multiplex Ligation-dependent Probe Amplification (MLPA) [16].

## 2. Case Description

A 34-year-old female diagnosed with 22q11.2DS was referred to our department for an ophthalmologic evaluation in May 2023. She complained of blurred vision and foreign body sensation at the presentation in both eyes. The patient denied any history of eye pain and ocular trauma. The family history was negative for genetic or ophthalmic diseases. The patient was diagnosed with 22q11.2DS following the recognition of ventricular septal defect, for which she underwent surgical repair shortly after birth. The diagnosis was confirmed by FISH. Her medical therapy consisted of bisoprolol for cardiac support. The patient applied no topical eye treatments.

**Figure 1:**
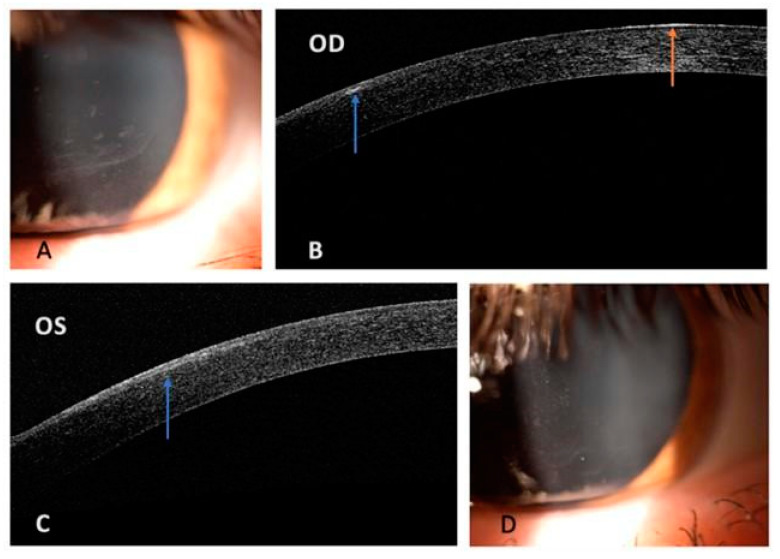
Patient’s slit-lamp and anterior segment optical coherence tomography images. (**A**) Slit-lamp photograph of the right eye showing elongated irregular opacities of the corneal epithelium. (**B**,**C**) Anterior segment optical coherence tomography scans of both eyes. The corneal stroma and the underlying layers have a normal aspect. (**B**) Presence of an abnormal spot of the epithelial basement membrane (blue arrow) and an irregular hyperreflective region of the corneal epithelium (orange arrow) in RE. (**C**) Evidence of diffuse thickening of the epithelial basement membrane, resulting in a large highly reflective area (blue arrow) involving the overlying epithelium in LE. (**D**) Slit-lamp photograph of the left eye demonstrating geographic map-like lines surrounding epithelial dots. RE: right eye; LE: left eye.

The best corrected visual acuity was 20/40 in the right eye (RE) and 20/20 in the left eye (LE), with a refraction of sph-4 cyl-1 ax 50° and sph-3.5 cyl-1 ax 150°, respectively. Slit lamp biomicroscopy showed the presence of bilateral inferior paracentral corneal lesions, suggestive of map-dot-fingerprint dystrophy (Figure 1). There was no corneal fluorescein staining. Posterior embryotoxon was also observed in both eyes. Additional exams were conducted to better characterize the corneal lesions, including Placido disk-based corneal topography, corneal pachymetry, specular microscopy, anterior segment optical coherence tomography, and in vivo confocal microscopy. Corneal topography was performed using the Sirius Scheimpflug–Placido disk topographer (Sirius, CSO, Firenze, Italy). The analysis of topographic maps showed no pathological alterations compatible with corneal ectasia. Specifically, the Kmax and Kmean values were 49.01 D (RE) and 48.73 D (LE), and 46.66 D (RE) and 46.74 D (LE), respectively. Additionally, no displacement of the thinnest point (490 μm RE and 498 μm LE) was observed, nor was there any correspondence with areas of posterior elevation. The anterior tangential map reveals irregularities in corneal curvature, likely attributable to changes in the epithelial basement membrane complex, leading to tear film instability. Overall, the exam showed no significant alterations, highlighting mild astigmatism in both eyes (Kast RE: −1.52 D; LE: −1.69 D). Central corneal thickness was 494 μm in the right eye and 499 μm in the left eye. The inferior peripheral corneal thickness at 8 mm on the pachymetry map was at the upper limits of normal in the right eye (710 μm RE; 658 μm LE). This finding may partially be attributable to the presence of a thickened basement membrane with an irregular overlying epithelial layer. In addition, specular microscopy (Perseus, CSO, Firenze, Italy) was performed, showing no alterations in the endothelial mosaic and no pathological changes in the endothelial cell density (RE: 2436 cells/mm^2^; LE: 2679 cells/mm^2^). Anterior segment optical coherence tomography (RTVue, Optovue Inc., Fremont, CA, USA) revealed small epithelial and subepithelial hyperreflective areas, with a thickening of the epithelial basement membrane and absence of stromal opacities (Figure 1). Both results support the suspicion of EBMD.

**Figure 2:**
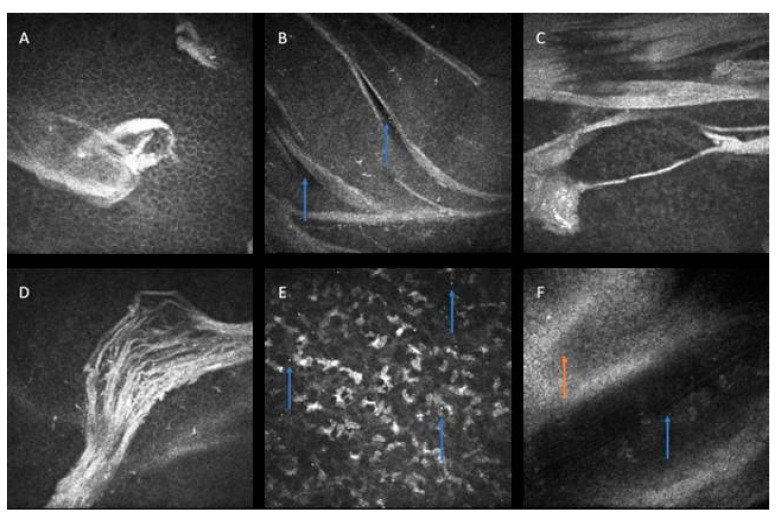
In vivo confocal microscopy images of EBMD-affected corneal layers. (**A**) Irregularly shaped extracellular hyperreflective deposits in the basal epithelium (depth 44 µm). (**B**) Hyporeflective lines (blue arrows) interspersed between highly reflective curvilinear changes in the basal epithelium (depth 46 µm). (**C**) Linear and ring-shaped highly reflective material in the epithelial basement membrane (depth 55 µm). (**D**) Sheetlike deposits in epithelial basement membrane (depth 63 µm). (**E**) Highly reflective microdots (blue arrows) spread in the anterior stroma (depth 82 µm). (**F**) Descemet membrane folds (orange arrow) and hyporeflective striae (blue arrow) in the posterior stroma (depth 465 µm). These findings may represent pressure-induced artifacts and should be distinguished from pathological changes.

Moreover, the patient underwent in vivo confocal microscopy (Heidelberg Engineering, Heidelberg, Germany) (Figure 2). Corneal map lines appeared as hyperreflective extracellular material located in the basal epithelium and Bowman’s membrane layer. The deposits were variously arranged in linear, rounded, geographic, and multilaminar patterns. Conversely, the fingerprints microscopically correspond to multiple hypo-reflective lines in the epithelium and Bowman’s membrane. In the same layers and the anterior stroma, microdots were detected as highly reflective spots. There were no abnormalities in the posterior stroma and the underlying layers.

**Figure 3:**
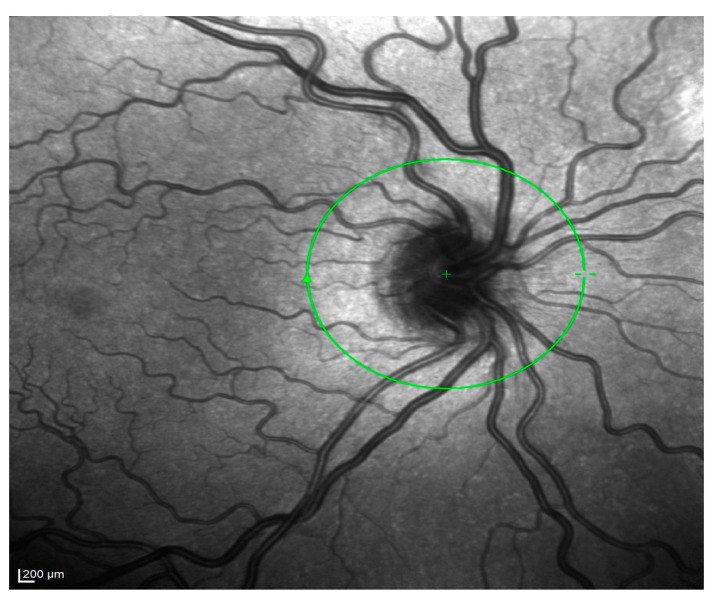
Near-infrared OCT of the posterior pole highlighting marked vascular tortuosity and tilted optic disk.

Finally, a dilated funduscopic examination showed significant bilateral retinal vascular tortuosity and a tilted optic disk (Figure 3). The rest of the fundus oculi was within normal limits.

## 3. Focus on EBMD

EBMD is the most frequent anterior corneal dystrophy, occurring in about 2–43% of individuals [17]. It is often referred to as map-dot-fingerprint dystrophy, as it typically presents with various combinations of geographic map-like lines, epithelial dots, and subepithelial fingerprints on slit-lamp examination. The map-like lines are represented by irregular, slightly grayish areas flanked by clearer zones, typically arranged around the epithelial dots or Cogan’s microcysts. Occasionally, fingerprint patterns are also observed in the same patient [18,19,20]. The basis of these alterations lies in an abnormal thickening of the Bowman’s membrane, which protrudes towards the corneal epithelium, determining the onset of opacities of variable shape and altering the normal desquamation of the epithelial cells, which remain trapped in the corneal layers, forming microcysts [18,21].

EBMD pathophysiology is still unclear. Since its prevalence increases with age, the latest IC3D classification of corneal dystrophies reported that the disease typically results from age-dependent corneal degeneration [22]. Nevertheless, in a minority of cases, a genetic role has been assessed, and some authors suggest a hereditary nature of the condition [23,24]. Recently, *TGFBI* (transforming growth factor b-induced) pathogenic variants have been identified as the cause of several corneal dystrophies, including EBMD. According to Boutboul et al. work, 10% of EBMD patients had *TGFBI* variants [23]. These data were subsequently validated by Evans et al., in which the incidence was 9%, although the mutations were different from those previously known [25]. The multiple TGFBI variants causative of EBMD highlight a genetic heterogeneity of the dystrophy and suggest the need for future research in this field. While the deletion of the 22q11.2 region is probably not causative for EBMD, our patient showed the presence of this genetic entity in both eyes, a first in the literature. The patient’s young age strongly indicates genetic etiology, since the epithelial basal membrane linked to degenerative causes is associated with old age. EBMD is associated with the mutation of TGFBI in chromosome 5, but not all the affected patients show the mutation. We can hypothesize that other genetic alterations can be causative for this clinical condition. 22q11.2DS is known to compromise many loci involved in systemic and ocular developmental pathways, and we can hypothesize that some of them can be responsible for EBDM. However, other causative genes for this corneal manifestation need to be identified.

The disease is usually asymptomatic; however, central lesions may lead to corneal astigmatism, resulting in blurred vision and photophobia [26]. Moreover, as the clinical case reported in the present study, up to 30% of patients experience recurrent episodes of ocular pain, foreign body sensation, and tearing, following spontaneous erosions of the corneal epithelium [27,28]. The symptoms are usually exacerbated on awakening due to nocturnal corneal dehydration. For this reason, hypertonic nighttime lubricants, tear substitutes, and/or therapeutic contact lenses may help prevent epithelial damage and reduce daytime symptoms. In addition, Labetoulle et al. proposed the use of topical heparan sulfate mimetic polymer to reduce ocular discomfort in patients with EBMD resistant to conventional symptomatic treatments, with a high success rate [29]. In cases of severe recurrent corneal erosions, epithelial debridement, anterior stromal puncture, and phototherapeutic keratectomy (PTK) is indicated [30,31,32,33,34].

The diagnosis is achieved by a careful slit-lamp examination. Recently, anterior segment optical coherence tomography and in vivo confocal microscopy have shown great usefulness in elucidating the morphologic alterations of EBMD and facilitated its diagnosis, especially in doubtful cases on slit lamp examination [35,36,37]. In particular, confocal microscopy allows for in vivo visualization of EBMD signs at the cellular level, enabling their differentiation from other similar basement membrane disorders. Except for the microcysts, all the distinguishing EBMD features have been documented in our patient extending the set of corneal manifestations already described in association with this syndrome [36,38,39]. As the genetic mechanisms underlying the dystrophy are still largely unexplained, further research is warranted to investigate a potential molecular basis between the two conditions.

## 4. Review of the Main Ocular Manifestations in 22q11.2 DS

Different studies and case reports highlighted the association between the 22q11.2DS and ocular pathologies over time even though the knowledge and frequency of eye involvement are still not well established.

### 4.1. Adnexa, External Eye Structures and Globe Involvement

A wide range of manifestations involving the ocular adnexa are reported in the recent literature on patients affected by 22q11.2DS.

Chandramohan et al. described a case of unilateral inferior orbital mass, responsible for motility abnormalities and eyelid alterations. Magnetic resonance imaging (MRI) was used to better characterize the lesion, which was defined as a congenital colobomatous intraorbital cyst involving the optic nerve and displacing the eyeball. The cystic lesion determined microphthalmia, restricted eye movement, and mechanical ectropion [40].

A single case report described a complete and bilateral lack of the inferior lacrimal ducts in association with the absence of the membranous nasolacrimal ducts in a 5-year-old child presenting 22q11.2DS. The patient underwent a bilateral endoscopic dacryocystorhinostomy. The authors concluded that due to the potential occurrence of the dysgenesis of the nasolacrimal duct in patients with systemic syndromes, symptoms such as mucous discharge or epiphora should be investigated thoroughly [41].

Moreover, cases of eyelid hooding and congenital ptosis have been identified. In 2005 a large study involving 240 patients affected by congenital heart diseases was conducted. The study aimed to collect ocular characteristics in association with cardiac anomalies. Among the cohort, 24 patients with 22q11.2DS were enrolled. The authors detected one case of Duane retraction syndrome and three cases of palpebral ptosis [42]. In another study conducted by Forbes et al. on 90 patients, eyelid hooding and ptosis were the most common findings involving the ocular adnexa. The authors suggested that eyelid hooding contributes to the facial feature of 22q11.2DS despite being present in 20% of the enrolled patients. Another relevant feature reported was a relatively high frequency of strabismus and motility disorders (18% compared to 4% of the general population). The most commonly reported feature was exotropia [43].

Eyelid hooding, narrow palpebral fissures, and floppy eyelids are also described by Gokturk et al. and Mansour AM et al. [42,44,45].

Microphthalmia is frequently described in the literature in association with this genetic condition, with bilateral or unilateral presentation. The reported cases include association with bilateral sclerocornea (one patient-one eye) [46], intraorbital cyst with a large chorioretinal coloboma, and complete chorioretinal coloboma, with visible retrolental persistent fetal vasculature (one patient-bilateral presentation) [40] and contralateral mild sclerocornea, corneal staphyloma, and congenital aphakia (one patient, one eye) [47]. These findings suggest that the genes responsible for anterior segment dysgenesis such as *CRYBB1*, *CRYBB2*, and *CRYBB3,* which are located in 22q11.2, may also influence the development of the whole globe [48].

### 4.2. Corneal Involvement

Corneal involvement in 22q11.2 DS is quite characteristic and presents multiple expressions. Keratoconus is a corneal ectasia characterized by a progressive thinning and steepening of the corneal stroma, which is commonly localized in the inferotemporal sector, but it can compromise the central region as well [49]. Different authors who described ophthalmological manifestation in 22q11.2 DS identified some cases of keratoconus [50,51,52].

Sclerocornea is a rare congenital condition presenting with different degrees of corneal opacification proceeding from the limbus to the central cornea. The cause of sclerocornea is attributable to a defective migration of neural crest cells determining a failure in the limbus development [53]. Sclerocornea has been described in patients affected by 22q11.2DS mostly in combination with other dysgenic features involving the anterior segment such as descemetocele, microphthalmia, corneal staphyloma, congenital aphakia, and iridocorneal adhesions [46,47].

Descemetocele is the protrusion of the Descemet membrane through a deep corneal stromal defect [54] and has been described as a single case series by Binenbaum and colleagues in five eyes of 5 patients with 22q11.2DS [46].

Corneal staphyloma is an extremely rare congenital condition encountered among the causes of congenital corneal opacification. This condition manifests with severe anterior corneal protrusion and opacification, often requiring penetrating keratoplasty [55]. Tarlan et al. reported a unilateral case of corneal staphyloma in a 2-year-old child affected by 22q11.2 DS. Fluorescence in situ hybridization analysis showed a heterozygous 250 kb region deletion in the 22q11.2DS critical region 2 [47]. Corneal manifestations are reported in Table 1.

### 4.3. Anterior Segment Involvement

The most commonly described dysgenetic feature in 22q11.2DS patients is posterior embryotoxon. It manifests with an anterior displacement of the Schwalbe’s line and the terminal portion of the Descemet membrane. Glaucoma represents one of the main complications affecting 50% of patients with embryotoxon due to concomitant iridogoniodysgenesis [56,57]. Many clinicians described the presence of posterior embryotoxon in consistent percentages of patients affected by 22q11.2DS. Among them, Mansour et al. identified posterior embryotoxon in 23% of their sample (five patients out of twenty-two) [45]. Casteels and coworkers evaluated 36 children affected by 22q11.2DS, and they identified posterior embryotoxon in 32 eyes [58]. Forbes et al. evaluated 90 patients with confirmed 22q11.2DS and described posterior embryotoxon in 44 cases (49% of the sample) [43]. Gokturk et al. identified posterior embryotoxon in 50% of their sample (eight patients), becoming one of the most diffuse alterations among their group of patients [44].

Aniridia is a congenital dysembryogenic disorder causing various grades of iris hypoplasia. It is usually related to autosomal dominant mutations involving the *PAX6* gene. This pathology can be associated with other ocular findings such as corneal opacification, glaucoma, or cataract [59]. In association with 22q11.2DS, researchers have described isolated cases of aniridia or iris hypoplasia. Moreover, bilateral iridocorneal adhesions or other severe anterior segment dysgenic anomalies such as crystalline lens congenital absence or lens subluxation have been reported [40,44,46,47].

Peters anomaly is a congenital malformation of the eye’s anterior segment, resulting from inherited or sporadic mutations in key developmental genes such as PAX6 [60]. Reis et al. described an 8-year-old female with 22q11.2DS who developed glaucoma at birth, alongside the Peters anomaly. Genetic testing revealed a heterozygous variant in the CYP1B1 gene, a common mutation associated with congenital and juvenile glaucoma [61]. Other reports also link Peters anomaly with 22q11.2DS. Erdogan et al. documented a 4-month-old with facial dysmorphism and right eye leukocoria. Slit lamp examination confirmed Peters anomaly and subsequent fluorescent in situ hybridization identified 22q11.2DS [62]. In a study by Casteels et al., one out of thirty-six young patients with 22q11.2DS had Peters anomaly [58]. Another case involved a 3-year-old with unilateral corneal opacity, where Peters anomaly was accompanied by peripheral corneal scleralization. The development of Peters anomaly in these patients is likely due to defective neural crest cell maturation [63]. Other particular features involving the iris within the framework of this pathology are iris coloboma, prominent iris processes, prominent iris crypts, iris remnants, and iris nodules [43,44,45,58]. Isolated congenital cataracts or lens opacities have also been described in a few papers [45,64]. Table 2 describes the alterations of the anterior segment most frequently associated with 22q11.2DS.

### 4.4. Posterior Segment Involvement

One of the most frequently reported features involving the posterior segment in this genetic condition is vascular retinal tortuosity also observed in our clinical case. Mansour et al. identified eight cases of retinal tortuosity in a cohort of twenty-two DiGeorge patients, and years later, eleven cases in a sample of twenty-four patients [42,45]. Casteels and coworkers evaluated 36 children affected by 22q11.2DS, and the posterior segment evaluation demonstrated retinal vessel tortuosity in 56 eyes (28 patients) [58]. Saffra et al. presented the case of a 23-year-old male patient affected by 22q11.2DS, and keratoconus, and additionally, the posterior segment examination revealed the presence of a tortuous retinal vascular tree [50]. A large group of 128 patients with 22q11.2DS were enrolled in 2016 by Kufert et al. The patients underwent a full-body clinical evaluation and a psychiatric assessment. The study had the purpose of collecting information about the variety of medical conditions and disorders affecting these patients. The clinicians identified ophthalmological involvement in 30.5% of the sample. They described tortuous retinal vessels in 3.9% of the sample (five patients) [52]. Forbes et al. in their study involving 90 patients with confirmed 22q11.2DS identified tortuous retinal vasculature in 31 subjects (34% of the sample) [43]. Several other works described this vascular anomaly in patients with this genetic condition [44,65,66]. Different optic disk anomalies have been recognized in this genetic condition, particularly optic disk atrophy, optic nerve drusen, and small or tilted optic disk as noted in the patients described above [42,43,44,45,60,66].

Chorioretinal coloboma is an ocular malformation occurring during embryogenesis, due to a failure in the closure of the embryonic fissure. This anomaly can develop as a sporadic manifestation with an autosomal dominant inheritance, or it can be familiar with autosomal recessive inheritance more commonly. Chorioretinal colobomas are also described in the context of systemic genetic diseases [67,68].

Many researchers described chorioretinal colobomas in patients with 22q11.2DS syndrome. Chandramohan et al., during the examination of a 4-month patient, identified an extensive chorioretinal coloboma compromising the posterior pole structures in the RE. The left eye (LE) was affected by a complete coloboma involving the retina and choroid. Moreover, the persistence of fetal retrolental vasculature was observed in the LE [40]. Midbari Kufert et al., in a large cohort of one hundred twenty-eight patients, described one case of chorioretinal coloboma [52]. The literature has also described the case report of a child affected by 22q11.2DS, presenting familial exudative vitreoretinopathy (FEVR), in the absence of the pathognomonic mutations *LRP5* and *FZD4* [69]. Many other sporadic alterations of the posterior segment in patients with 22q11.2DS are reported in the literature and summarized in Table 3.

## 5. Conclusions

In conclusion, we report the first case of coincident EBMD and 22q11.2DS in a young adult patient. Several papers reported extensive case series of systemic manifestations including ocular features in childhood. However, there is poor evidence of potential new manifestations that may develop in the adult population. It seemed interesting to report this association, which has never been described before in the literature, even though it is not possible to determine if there is a connection between the two conditions. Given that EBMD typically develops between the third and sixth decades of life, it would be suggested that patients with 22q11.2DS undergo regular ocular examinations until adulthood to promptly identify and manage corneal and ocular changes.

## Figures and Tables

**Table 1:** Corneal manifestations and relative frequency. The sclerocornea frequency appears higher than expected as Binenbaum et al. only recruited patients affected by sclerocornea [46].

Manifestation	Number of Patients	Author
Keratoconus	1 (case report)	Saffra et al., 2015 [50]
	1 out of 10	Bassett et al., 1998 [51]
	1 out of 128	Midbari et al., 2016 [52]
Sclerocornea	7 (100%)	Binenbaum et al., 2008 [46]
	1 (case report)	Tarlan et al., 2014 [47]
Corneal staphyloma	1 (case report)	Tarlan et al., 2014 [47]
Descemetocele	3 (42.8%)	Binenbaum et al., 2008 [46]

**Table 2:** Anterior segment manifestations and relative frequency. * The number expresses eyes, not patients.

Manifestation	Number of Patients	Author
Posterior embryotoxon	44 (49%)	Forbes et al., 2007 [43]
	8 (50%)	Gokturk et al., 2016 [44]
	5 (23%)	Mansour et al., 1987 [45]
	32 (44%) *	Casteels et al., 2008 [58]
Peters anomaly	1 (out of 36)	Casteels et al., 2008 [58]
	3 (case reports)	Reis et al., 2015 [61]; Erdogan et al., 2008 [62]; Casteels et al., 2005 [63]
Aniridia	1 (case report)	Chandramohan et al., 2021 [40]
Lens subluxation	1 (case report)	Chandramohan et al., 2021 [40]
Congenital aphakia	1 (case report)	Tarlan et al., 2014 [47]
Cataract	1 out of 22	Mansour et al., 1987 [45]
	1 (case report)	Allegrini et al., 2017 [64]
	1 out of 36	Casteels et al., 2008 [58]
Iridocorneal adhesions	1 out of 7	Binenbaum et al., 2008 [46]
Severe anterior segment dysgenesis	1 out of 7	Binenbaum et al., 2008 [46]
	2 (case reports)	Erdogan et al., 2008 [62]; Tarlan et al., 2014 [47]

**Table 3:** Posterior segment manifestations and relative frequency.

Manifestation	Number of Patients	Author
Retinal vascular tortuosity	11 (61%)	Mansour et al., 2005 [42]
	31 (34%)	Forbes et al., 2007 [43]
	9 (56.2%)	Gokturk et al., 2016 [44]
	8 (36.3%)	Mansour et al., 1987 [45]
	4 (case reports)	Saffra et al., 2015 [50]; Kozak et al., 2022 [69]; Allegrini et al., 2017 [64]; De Niro et al., 2013 [65]
	5 (3.9%)	Midbari et al., 2016 [52]
	28 (77.7%)	Casteels et al., 2008 [58]
	8 (80%)	Crewther et al., 1998 [66]
Disk drusen	1 out of 24	Mansour et al., 2005 [42]
	1 (case report)	Allegrini et al., 2017 [64]
Chorioretinal colobomas	1 (case report)	Chandramohan et al., 2021 [40]
	1 out of 128	Midbari et al., 2016 [52]
Retrolental persistent fetal vasculature	1 (case report)	Chandramohan et al., 2021 [40]
Familial exudative vitreoretinopathy	1 (case report)	Gilmour et al., 2009 [69]
Diffuse retinal, vitreous, and papillar hemorrhages with vascular dysplasia	1 (case report)	Kozak et al., 2022 [70]
Retinopathy of prematurity	1 (case report)	Paulus et al., 2013 [71]
Optic disk hypoplasia	6 (33%)	Mansour et al., 2005 [42]
	1 out of 16	Gokturk et al., 2016 [44]
	4 (18%)	Mansour et al., 1987 [45]
	3 (30%)	Crewther et al., 1998 [66]
Tilted optic nerve	1 out of 90	Forbes et al., 2007 [43]
Optic disk swelling	1 (case report)	Girgis et al., 2004 [72]

## Data Availability

Not applicable.

## References

[1] Oskarsdóttir S., Vujic M., Fasth A. (2004). Incidence and prevalence of the 22q11 deletion syndrome: A population-based study in Western Sweden. Arch. Dis. Child..

[2] Shprintzen R.J. (2008). Velo-cardio-facial syndrome: 30 Years of study. Dev. Disabil. Res. Rev..

[3] Driscoll D.A., Salvin J., Sellinger B., Budarf M.L., McDonald-McGinn D.M., Zackai E.H., Emanuel B.S. (1993). Prevalence of 22q11 microdeletions in DiGeorge and velocardiofacial syndromes: Implications for genetic counselling and prenatal diagnosis. J. Med. Genet..

[4] McDonald-McGinn D.M., Sullivan K.E. (2011). Chromosome 22q11.2 deletion syndrome (DiGeorge syndrome/velocardiofacial syndrome). Medicine.

[5] Morrow B.E., McDonald-McGinn D.M., Emanuel B.S., Vermeesch J.R., Scambler P.J. (2018). Molecular genetics of 22q11.2 deletion syndrome. Am. J. Med. Genet. A.

[6] Papangeli I., Scambler P. (2013). The 22q11 deletion: DiGeorge and velocardiofacial syndromes and the role of TBX1. Wiley Interdiscip. Rev. Dev. Biol..

[7] Funato N. (2022). Craniofacial Phenotypes and Genetics of DiGeorge Syndrome. J. Dev. Biol..

[8] Shprintzen R.J., Goldberg R.B., Lewin M.L., Sidoti E.J., Berkman M.D., Argamaso R.V., Young D. (1978). A new syndrome involving cleft palate, cardiac anomalies, typical facies, and learning disabilities: Velo-cardio-facial syndrome. Cleft. Palate J..

[9] Wulfsberg E.A., Leana-Cox J., Neri G. (1996). What’s in a name? Chromosome 22q abnormalities and the DiGeorge, velocardiofacial, and conotruncal anomalies face syndromes. Am. J. Med. Genet..

[10] McDonald-McGinn D.M., Sullivan K.E., Marino B., Philip N., Swillen A., Vorstman J.A.S., Zackai E.H., Emanuel B.S., Vermeesch J.R., Morrow B.E. (2015). 22q11.2 deletion syndrome. Nat. Rev. Dis. Primers.

[11] Bassett A.S., Chow E.W.C., Husted J., Weksberg R., Caluseriu O., Webb G.D., Gatzoulis M.A. (2005). Clinical features of 78 adults with 22q11 Deletion Syndrome. Am. J. Med. Genet. A.

[12] Murphy K.C., Jones L.A., Owen M.J. (1999). High rates of schizophrenia in adults with velo-cardio-facial syndrome. Arch. Gen. Psychiatry.

[13] Gothelf D., Schaer M., Eliez S. (2008). Genes, brain development and psychiatric phenotypes in velo-cardio-facial syndrome. Dev. Disabil. Res. Rev..

[14] Rizvi S., Khan A.M., Saeed H., Aribara A.M., Carrington A., Griffiths A., Mohit A. (2018). Schizophrenia in DiGeorge Syndrome: A Unique Case Report. Cureus.

[15] Schneider M., Debbané M., Bassett A.S., Chow E.W.C., Fung W.L.A., Bree M.B.M.v.D., Owen M., Murphy K.C., Niarchou M., Kates W.R. (2014). Psychiatric disorders from childhood to adulthood in 22q11.2 deletion syndrome: Results from the International Consortium on Brain and Behavior in 22q11.2 Deletion Syndrome. Am. J. Psychiatry.

[16] Cortés-Martín J., Peñuela N.L., Sánchez-García J.C., Montiel-Troya M., Díaz-Rodríguez L., Rodríguez-Blanque R. (2022). Deletion Syndrome 22q11.2: A Systematic Review. Children.

[17] Werblin T.P., Hirst L.W., Stark W.J., Maumenee I.H. (1981). Prevalence of map-dot-fingerprint changes in the cornea. Br. J. Ophthalmol..

[18] Cogan D.G., Donaldson D.D., Kuwabara T., Marshall D. (1964). Microcystic Dystrophy of the Corneal Epithelium. Trans. Am. Ophthalmol. Soc..

[19] Guerry D. (1965). Observations on Cogan’s microcystic dystrophy of the corneal epithelium. Trans. Am. Ophthalmol. Soc..

[20] Trobe J.D., Laibson P.R. (1972). Dystrophic changes in the anterior cornea. Arch. Ophthalmol..

[21] Rodrigues M.M., Fine B.S., Laibson P.R., Zimmerman L.E. (1974). Disorders of the corneal epithelium. A clinicopathologic study of dot, geographic, and fingerprint patterns. Arch. Ophthalmol..

[22] Møller H.U., Weiss J.S. (2011). IC3D classification of corneal dystrophies. Dev. Ophthalmol..

[23] Boutboul S., Black G.C.M., Moore J.E., Sinton J., Menasche M., Munier F.L., Laroche L., Abitbol M., Schorderet D.F. (2006). A subset of patients with epithelial basement membrane corneal dystrophy have mutations in TGFBI/BIGH3. Hum. Mutat..

[24] Laibson P.R., Krachmer J.H. (1975). Familial occurrence of dot (microcystic), map, fingerprint dystrophy of the cornea. Investig. Ophthalmol..

[25] Evans C.J., Davidson A.E., Carnt N., López K.E.R., Veli N., Thaung C.M., Tuft S.J., Hardcastle A.J. (2016). Genotype-Phenotype Correlation for TGFBI Corneal Dystrophies Identifies p.(G623D) as a Novel Cause of Epithelial Basement Membrane Dystrophy. Investig. Ophthalmol. Vis. Sci..

[26] Ehlers N., Møller H.U. (1987). Dot-map-fingerprint dystrophy--Cogan’s microcystic dystrophy--normal reactions of the corneal epithelium?. Acta Ophthalmol..

[27] Laibson P.R. (2010). Recurrent corneal erosions and epithelial basement membrane dystrophy. Eye Contact Lens.

[28] Hykin P.G., Foss A.E., Pavesio C., Dart J.K. (1994). The natural history and management of recurrent corneal erosion: A prospective randomised trial. Eye.

[29] Labetoulle M., Rousseau A., M’Garrech M., Kaswin G., Dupas B., Baudouin C., Barreau E., Bourcier T., Chiambaretta F. (2019). Efficacy of a Topical Heparan Sulfate Mimetic Polymer on Ocular Surface Discomfort in Patients with Cogan’s Epithelial Basement Membrane Dystrophy. J. Ocul. Pharmacol. Ther..

[30] Orndahl M.J., Fagerholm P.P. (1998). Phototherapeutic keratectomy for map-dot-fingerprint corneal dystrophy. Cornea.

[31] Lee W.S., Lam C.K., Manche E.E. (2017). Phototherapeutic keratectomy for epithelial basement membrane dystrophy. Clin. Ophthalmol..

[32] McLean E.N., MacRae S.M., Rich L.F. (1986). Recurrent erosion. Treatment by anterior stromal puncture. Ophthalmology.

[33] Pogorelov P., Langenbucher A., Kruse F., Seitz B. (2006). Long-term results of phototherapeutic keratectomy for corneal map-dot-fingerprint dystrophy (Cogan-Guerry). Cornea.

[34] Vo R.C., Chen J.L., Sanchez P.J., Yu F., Aldave A.J. (2015). Long-Term Outcomes of Epithelial Debridement and Diamond Burr Polishing for Corneal Epithelial Irregularity and Recurrent Corneal Erosion. Cornea.

[35] Ramos J.L.B., Li Y., Huang D. (2009). Clinical and research applications of anterior segment optical coherence tomography—A review. Clin Exp. Ophthalmol..

[36] Hernández-Quintela E., Mayer F., Dighiero P., Briat B., Savoldelli M., Legeais J.-M., Renard G. (1998). Confocal microscopy of cystic disorders of the corneal epithelium. Ophthalmology.

[37] El Sanharawi M., Sandali O., Basli E., Bouheraoua N., Ameline B., Goemaere I., Georgeon C., Hamiche T., Borderie V., Laroche L. (2015). Fourier-domain optical coherence tomography imaging in corneal epithelial basement membrane dystrophy: A structural analysis. Am. J. Ophthalmol..

[38] Rosenberg M.E., Tervo T.M., Petroll W.M., Vesaluoma M.H. (2000). In vivo confocal microscopy of patients with corneal recurrent erosion syndrome or epithelial basement membrane dystrophy. Ophthalmology.

[39] Kobayashi A., Yokogawa H., Sugiyama K. (2012). In vivo laser confocal microscopy findings in patients with map-dot-fingerprint (epithelial basement membrane) dystrophy. Clin. Ophthalmol..

[40] Chandramohan A., Sears C.M., Huang L.C., Beres S., Fredrick D., Kossler A.L. (2021). Microphthalmia and orbital cysts in DiGeorge syndrome. J. AAPOS.

[41] Prabhakaran V.C., Davis G., Wormald P.J., Selva D. (2008). Congenital absence of the nasolacrimal duct in velocardiofacial syndrome. J. AAPOS.

[42] Mansour A.M., Bitar F.F., Traboulsi E.I., Kassak K.M., Obeid M.Y., Megarbane A., I Salti H. (2005). Ocular pathology in congenital heart disease. Eye.

[43] Forbes B.J., Binenbaum G., Edmond J.C., DeLarato N., McDonald-McGinn D.M., Zackai E.H. (2007). Ocular findings in the chromosome 22q11.2 deletion syndrome. J. AAPOS.

[44] Gokturk B., Topcu-Yilmaz P., Bozkurt B., Yildirim M.S., Guner S.N., Sayar E.H., Reisli I. (2016). Ocular Findings in Children With 22q11.2 Deletion Syndrome. J. Pediatr. Ophthalmol. Strabismus.

[45] Mansour A.M., Goldberg R.B., Wang F.M., Shprintzen R.J. (1987). Ocular findings in the velo-cardio-facial syndrome. J. Pediatr. Ophthalmol. Strabismus.

[46] Binenbaum G., McDonald-McGinn D.M., Zackai E.H., Walker B.M., Coleman K., Mach A.M., Adam M., Manning M., Alcorn D.M., Zabel C. (2008). Sclerocornea associated with the chromosome 22q11.2 deletion syndrome. Am. J. Med. Genet. A.

[47] Tarlan B., Kiratli H., Kılıç E., Utine E., Boduroğlu K. (2014). A case of 22q11.2 deletion syndrome with right microphthalmia and left corneal staphyloma. Ophthalmic Genet..

[48] Mataftsi A., Islam L., Kelberman D., Sowden J.C., Nischal K.K. (2011). Chromosome abnormalities and the genetics of congenital corneal opacification. Mol. Vis..

[49] Santodomingo-Rubido J., Carracedo G., Suzaki A., Villa-Collar C., Vincent S.J., Wolffsohn J.S. (2022). Keratoconus: An updated review. Cont. Lens Anterior. Eye.

[50] Saffra N., Reinherz B. (2015). Keratoconus in an adult with 22q11.2 deletion syndrome. BMJ Case Rep..

[51] Bassett A.S., Hodgkinson K., Chow E.W., Correia S., Scutt L.E., Weksberg R. (1998). 22q11 deletion syndrome in adults with schizophrenia. Am. J. Med. Genet..

[52] Midbari Kufert Y., Nachmani A., Nativ E., Weizman A., Gothelf D. (2016). Association between prematurity and the evolution of psychotic disorders in 22q11.2 deletion syndrome. J. Neural Transm..

[53] Quiroz-Casian N., Chacon-Camacho O.F., Barragan-Arevalo T., Nava-Valdez J., Lieberman E., Salgado-Medina A., Navas A., Graue-Hernandez E.O., Zenteno J.C. (2018). Sclerocornea-Microphthalmia-Aphakia Complex: Description of Two Additional Cases Associated With Novel FOXE3 Mutations and Review of the Literature. Cornea.

[54] Agarwal R., Nagpal R., Todi V., Sharma N. (2021). Descemetocele. Surv. Ophthalmol..

[55] Wan Y., Xiao G., Yu T., Zhang P., Hong J. (2020). Histopathological examination of congenital corneal staphyloma and prognosis after penetrating keratoplasty. Medicine.

[56] Rennie C.A., Chowdhury S., Khan J., Rajan F., Jordan K., Lamb R.J., Vivian A.J. (2005). The prevalence and associated features of posterior embryotoxon in the general ophthalmic clinic. Eye.

[57] Michels K., Bohnsack B.L. (2023). Ophthalmological Manifestations of Axenfeld-Rieger Syndrome: Current Perspectives. Clin. Ophthalmol..

[58] Casteels I., Casaer P., Gewillig M., Swillen A., Devriendt K. (2008). Ocular findings in children with a microdeletion in chromosome 22q11.2. Eur. J. Pediatr..

[59] Landsend E.C.S., Lagali N., Utheim T.P. (2021). Congenital aniridia—A comprehensive review of clinical features and therapeutic approaches. Surv. Ophthalmol..

[60] Bhandari R., Ferri S., Whittaker B., Liu M., Lazzaro D.R. (2011). Peters anomaly: Review of the literature. Cornea.

[61] Reis L.M., Tyler R.C., Zori R., Burgess J., Mueller J., Semina E.V. (2015). A case of 22q11.2 deletion syndrome with Peters anomaly, congenital glaucoma, and heterozygous mutation in CYP1B1. Ophthalmic Genet..

[62] Erdoğan M.K., Utine G.E., Alanay Y., Aktaş D. (2008). Unilateral Peters’ anomaly in an infant with 22q11.2 deletion syndrome. Clin. Dysmorphol..

[63] Casteels I., Devriendt K. (2005). Unilateral Peters’ anomaly in a patient with DiGeorge syndrome. J. Pediatr. Ophthalmol. Strabismus.

[64] Allegrini D., Penco S., Pece A., Autelitano A., Montesano G., Paci S., Montanari C., Maver A., Peterlin B., Damante G. (2017). Cataract and optic disk drusen in a patient with glycogenosis and di George syndrome: Clinical and molecular report. BMC Ophthalmol..

[65] De Niro J.E., Randhawa S., McDonald H.R. (2013). Retinal vascular tortuosity in DiGeorge syndrome complicated by solar retinopathy. Retin Cases Brief Rep..

[66] Crewther S.G., Kiely P.M., Kok L.L., Crewther D.P. (1998). Anomalies of genetic development as predictors of oculo-visual abnormalities in velo-cardio-facial syndrome. Optom. Vis. Sci..

[67] Uhumwangho O.M., Jalali S. (2014). Chorioretinal coloboma in a paediatric population. Eye.

[68] Pagon R.A. (1981). Ocular coloboma. Surv. Ophthalmol..

[69] Gilmour D.F., Downey L.M., Sheridan E., Long V., Bradbury J., Inglehearn C.F., Toomes C. (2009). Familial exudative vitreoretinopathy and DiGeorge syndrome: A new locus for familial exudative vitreoretinopathy on chromosome 22q11.2?. Ophthalmology.

[70] Kozak I., Ali S.A., Wu W.C. (2022). Novel retinal observations in a child with DiGeorge (22q11.2 deletion) syndrome. Am. J. Ophthalmol. Case Rep..

[71] Paulus Y.M., Moshfeghi D.M. (2013). Persistent plus disease after laser in retinopathy of prematurity with tetralogy of Fallot. Eur. J. Ophthalmol..

[72] Girgis R.a.M., McKee H.D.R., Innes J.R. (2004). Swollen optic discs in a patient with the chromosome 22q11.2 deletion syndrome. Br. J. Ophthalmol..

